# How to Secure Efficient Surgery

**Published:** 1918-01-26

**Authors:** 


### THE WAR AND ORTHOPÆDICS.

### How to Secure Efficient Surgery.

Owing to a mistaken etymology there is a widespread popular impression that orthopædics is an art which is concerned with the correction of deformities of the feet, whereas a true analysis—as also the history of the word—indicates that orthopædics has for its mission the making of "straight children." The practice at the outset, indeed, was devoted to such deformities as club-foot and curvature of the spine, and the means employed were largely mechanical, as splints and supports, in contrast to surgical operations in which the knife is a prominent factor. But, strictly speaking, an operation for hare-lip or cleft palate, or indeed for any of the deformities met with in children as a result of developmental defect, is an exercise in orthopædics. It is an attempt to "make straight" a crooked or deformed child. By extension, though the proceeding is an etymological outrage, the word gradually came to be applied also to the correction of deformities in adults, and at the present day its application is independent of the age of the patient. But even yet, in practice, the term implies that the methods adopted are physical and mechanical rather than surgical and operative, though these latter are not wholly excluded, and its use suggests proceedings which have for their aim conservation rather than destruction or removal. Orthopædics, in short, seeks to overcome deformities and to neutralise defects by the institution of compensation. It is, of course, a department of the surgical art, but it is conservative, not radical surgery.

With such methods and aims it is little wonder that orthopædic surgery has been brought into great prominence by certain of the inevitable events of the war. Stiff joints, wasted muscles, damaged nerves, deformed and amputated limbs, and similar conditions, are unhappily only too numerous; they are indeed to be numbered by thousands. In some instances, given proper treatment, the sufferer can be restored to the fighting ranks of the Army; in others, although military value is permanently destroyed, the man can be fitted for some civil occupation, and can thus be saved from the dangers of an idle and purposeless life and given a useful status in the community. The orthopædic surgeon is therefore not in want of a worthy sanction for his work. Now, in the nature of things, this work goes outside what may be called the rigid lines of professional procedure, as for practical purposes it is not always enough that a stiff joint be rendered flexible or that a bent limb be made straight. What is to be desired is that the man's limbs be of use to him for work in the world and for the earning of his livelihood. If happily he can be restored to the same level and degree of efficiency as he enjoyed prior to his injury, well and good. But when this is not possible he may still be equipped for some order of work, and thus be redeemed for the nation. But obviously in such instances educational training is necessary, and it often happens that such training may be made an effective aid to the restoration of function. Hence it is impossible to set up some hard-and-fast line which will divide the treatment of the case into a strictly surgical part, to be conducted by the orthopædic surgeon, and an educational part, to be directed by some other authority. The two areas overlap, and not infrequently the two aims are served by one and the same means. Some one and the same authority must survey and direct the whole undertaking, and must co-ordinate the means to be employed in order that by wise adjustment there may be obtained both a re-established function and the application of this to a determined and serviceable purpose. It is manifest that work of this order possesses a very special quality, and the judgment necessary to undertake it with success can only come from a well-utilised experience. Hitherto the amount of such work has been a very limited quantity, and the practitioners who have devoted themselves to it have been few in number. In consequence of the war the cases requiring orthopædic treatment are legion, and the doctors needed to meet such a position simply do not exist. They have to be created, and the necessary training is not to be accomplished in a day. Nor, be it noted, is the work necessarily attractive to all who cultivate a distinctly surgical bent. It lacks the dramatic appeal of the brilliant operation, and pursues its ends by methods which require much patience and perseverance. Gradually the medical profession is being led to a recognition of the actual position, and time will doubtless yield the necessary help.

Much has already been accomplished by Colonel Sir Robert Jones, who has for long occupied a pre-eminent position in this field of work. In a recent address Colonel Jones has related both the urgency of the situation and the means by which he proposes to meet it. Military hospitals for orthopædic treatment now exist at many centres, and it is manifest that these must be used as training-schools for the surgeons needed to deal with the situation. Further, and of the greatest importance, the surgeons at the Front must learn from their orthopædic colleagues what are the most suitable methods of treating certain injuries, in order that subsequent restorative and educational treatment may command the largest success. Orthopædic surgery must be in view from the outset, and must not merely be regarded as a convenient refuge for cases in which other methods have failed. Never before has this branch of surgery had such multiplied opportunities, and we are satisfied that under the existing direction it will not prove itself unequal to the occasion. There must be an established relation between orthopædics and general surgery, and a proportion of the younger men must be enlisted for the training necessary to the development of the orthopædic expert.

